# Addressing malnutrition in hemodialysis: the potential role of serum transferrin as a biomarker and the need for a multi-marker approach

**DOI:** 10.1080/0886022X.2024.2350237

**Published:** 2024-05-07

**Authors:** Rayyan Nabi, Tabeer Zahid, Hanzala Ahmed Farooqi

**Affiliations:** a Islamic International Medical College, Rawalpindi, Pakistan; b Foundation University Medical College, Islamabad, Pakistan

Dear editor,

Chronic kidney disease has posed a significant health burden in the last three decades, and hemodialysis (HD) has emerged as the mainstay of treatment in 89% of the patients with end stage kidney disease (ESKD). However, a crucial problem encountered by these patients is that of malnutrition, with a global prevalence of 28–54% [1]. Not only does this adversely affect quality of life, but also increases risk of infections, thereby increasing mortality. Thus, effective strategies to timely detect and treat malnutrition and related problems in HD patients is of utmost importance to improve clinical outcomes.

For this reason, Benjamin et al. conducted a prospective study on 59 patients with ESKD on HD to assess whether serum transferrin could be used as a potential biomarker for malnutrition in the HD population [2]. Anthropometric measurements (height, weight, mid upper arm circumference (MUAC), and skinfold thickness) and blood tests for hemoglobin (Hb), transferrin (Tfr), albumin (Alb), iron (Fe), and ferritin (Ferr) were done on a monthly basis for six months. The data obtained was analyzed to identify associations between the selected clinical and laboratory parameters. Analysis of the clinical parameter MUAC showed a significant association with transferrin levels only; on the other hand, laboratory parameter transferrin level analysis revealed significant associations with MAUC, skinfold thickness, albumin, and dry weight. Neither MUAC nor transferrin levels were found to have a significant association with hemoglobin levels. As a result, the study demonstrated a strong link between low transferrin levels (< 2.15 g/dL to 3.80 g/dL) and malnutrition, with 97.7% of HD patients falling into one of the malnutrition groups.

According to Benković et al. adult malnourished patients with a few selected chronic diseases incur an annual cost of around 97 million euros. It was also observed that undernutrition was commonly observed (65%) in patients with chronic renal failure undergoing HD [3]. Since transferrin levels can be measured by simple serum-based tests which are often a part of routine blood workup, their use as a potential marker for early detection and subsequent intervention in malnourished patients undergoing HD may have serious merits. The development of such a patient-centered approach, along with benefitting patients, will also aid in preventing excessive waste of healthcare resources. This will help mitigate the enormous economic burden of malnutrition, especially in developing countries. However, a contradictory opinion suggests that biochemical parameters like transferrin, hemoglobin, prealbumin and serum creatinine etc., when used solely, are insensitive diagnostic markers of malnutrition [4]. It is our view, that with transferrin also being a marker of Iron stores among others, it may not be wise to use it as a determinant of malnutrition – a condition with many facets – on its own. Therefore, further studies investigating the potential of a multi-marker approach by integrating clinical parameters like anthropometric measurements as well as biochemical parameters are needed, potentially discovering a ground-breaking strategy for effective diagnosis. We also believe certain other aspects of the study by Benjamin et al. pose limitations and further studies in the future are needed to address these concerns. Firstly, the small sample size calls for multi-center studies with a larger group of subjects so that the accuracy of results may be improved. Moreover, given that the authors’ findings are from a very specific patient population of South Africa, we recommend similar studies on a more diverse group of patients in order to negate any difference in results that may have occurred due to race or ethnicity. Doing so may enable the inclusion of positive results in clinical guidelines and pave the way for their wide-scale implementation in clinical settings.
